# Endoplasmic Reticulum Membrane and Contact Site Dynamics in Autophagy Regulation and Stress Response

**DOI:** 10.3389/fcell.2020.00343

**Published:** 2020-05-29

**Authors:** Etienne Morel

**Affiliations:** Cell Biology Department, Institut Necker-Enfants Malades (INEM), INSERM U1151-CNRS UMR 8253, Université de Paris, Paris, France

**Keywords:** ER, autophogosome, membrane contact site, lipids, biogenesis

## Abstract

Autophagy mobilizes a variety of intracellular endomembranes to ensure a proper stress response and the maintenance of cellular homeostasis. While the process of *de novo* biogenesis of pre-autophagic structures is not yet fully characterized, the role of the endoplasmic reticulum (ER) appears to be crucial in early steps of autophagic process. Here, I review and discuss various aspects of ER and ER-driven membrane contact site requirements and effects on mammalian organelles and endomembrane biogenesis, in particular during the early steps of autophagy-related membrane dynamics.

## The Molecular Mechanisms of Autophagy and Autophagosome Biogenesis

Macroautophagy (hereafter referred to as autophagy) is an evolutionarily conserved intracellular catabolic pathway that ensures degradation, turnover, and renewal of intracellular and cytosolic components. Autophagy necessitates the formation of a double-membrane organelle termed the autophagosome that ensures the capture and the transport of cargoes to the acidic lysosome (Boya et al., 2013). Autophagy functions in most mammalian cells at low levels, a condition commonly referred to as basal autophagy. However, a stimulated autophagy response can be induced in response to stress-related situations, such as nutrient(s) deprivation(s), infection, physical, or mechanical or chemical stresses. Because autophagic process is a crucial cell-survival mechanism and a pivotal cellular homeostasis regulator, it has been studied for decades in physiological conditions and disease (Yang and Klionsky, 2010). Defects in autophagy have been associated with a variety of human diseases such as cancer, inflammation, neurodegenerative diseases, and metabolic disorders (Boya et al., 2013).

**FIGURE 1:**
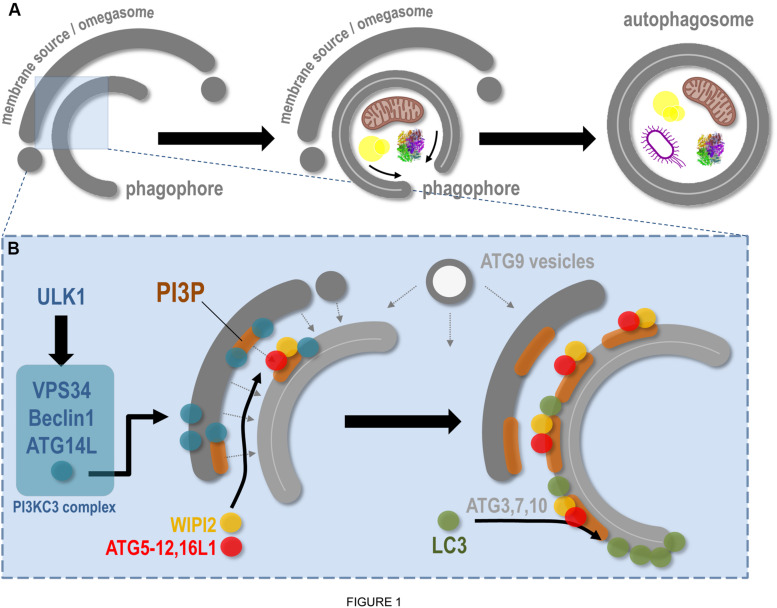
Molecular aspects of phagophore biogenesis and autophagy initiation. This scheme summarizes in a simplified way the main steps associated with membrane remodeling events leading to phagophore assembly. **(A)** The *de novo* biogenesis of the pre-autophagosomal phagophore (also called the isolation membrane) occurs at the ER-associated omegasome and membrane(s) source(s) interface. The phagophore maturation implies cargoes (specific, such as mitochondria, lipid droplets, protein aggregates, bacteria, etc., and non-specific) capture, physical disassembly from the membrane source, and closure, through fission of limiting membrane, which leads to double membrane autophagosome formation. **(B)** At the omegasome and membrane source interface, the stress-induced ULK1 autophagic complex is locally recruited and in turn allows the direct activation and membrane binding of the PI3KC3 complex, notably composed of VPS34 (the lipid kinase), Beclin1, and ATG14L. Membrane fueling and *de novo* assembly initiate future phagophore biogenesis, via membrane(s) and lipid delivery (dashed arrows), including lipids from ATG9-positive vesicles. Concomitantly, the presence of VPS34 leads to PI3P local synthesis, a necessary step for membrane flagging and for major ATG recruitment to pre-autophagosomal membrane. Via interaction with the PI3P-binding WIPI2, and via a direct anchoring to PI3P-positive membranes, the ATG16L1 master regulator allows the targeting of the ATG5–12 complex to the membrane, which in turn, with the help of cytosolic ATGs, promotes the local lipidation of LC3 protein at the surface of the future phagophore.

The stimulated autophagic response, notably induced by nutrient deprivation, requires several key steps that will lead eventually to cytoplasmic material (such as protein aggregates, pathogens, or damaged organelles) sequestration by a newly formed autophagosome and delivery to the lysosome for degradation. This dynamic sequence of events first requires complex signalization that will allow the specific mobilization of dedicated proteins, lipids, and membranes to ensure the formation and the maturation of the autophagosome; its transport inside the cytoplasm; and its fusion with lysosome. Autophagosome biogenesis starts with the assembly of a pre-autophagosomal cup-shaped membrane, the phagophore or isolation membrane, which captures autophagic cargoes and closes up to form a mature autophagosome (Figure 1). Most of these membrane-related events are regulated by autophagy-related genes (ATG) proteins, with non-ATG partners mostly required for intracellular signaling and membrane transportation on trafficking platforms (Walker and Ktistakis, 2019). The origin of the phagophore is still largely unknown (see section “The ER Membrane and ER Contact Sites in Autophagy Regulation”). It is suggested that this transient structure emanates from multiple origins, such as endosomal and Golgi vesicles, mitochondria, and the ER itself.

While there are several signaling pathways involved in autophagy, the mTORC1 and AMPK protein complexes appear to be crucial for the mobilization of the autophagic machinery (Molino et al., 2017b) in many stress situations. Inhibition of the mTORC1 signaling pathway leads to the activation of the ULK1 complex [composed of ULK1/2 kinases (ATG1/2), FIP200, ATG13, and ATG101]. The ULK1 complex will in turn activate the lipid kinase class III PI3K (formed by VPS34, Beclin1, VPS15, and ATG14L1) which generates the production of phosphatidylinositol-3-phosphate (PI3P) locally on ER subdomains, known as the pre-autophagosomal membrane(s) or omegasomes (Figure 1; Tooze, 2013).

The pre-autophagosomal membrane(s) are hallmarked by a dedicated pool of PI3P required for phagophore formation and expansion. PI3P allows the recruitment of several ATG proteins, including members of the WIPI family. WIPI2, which binds to PI3P via a proppins domain (Baskaran et al., 2012), recruits the ATG16L1–ATG5/12 conjugation system to the pre-autophagosome (Dooley et al., 2014), allowing in turn the membrane targeting of the LC3 protein (yeast ATG8 homolog), so far considered as the bona fide marker of autophagic organelles (Figure 1; Nishimura et al., 2013; Dooley et al., 2014; Wilson et al., 2014). Recently the autophagosome biogenesis key regulator ATG16L1 was shown to bind also to PI3P (Dudley et al., 2019), further stabilizing the conjugation complex. LC3 recruitment to the future autophagosome occurs via its lipidation (via the adjunction of a PE moiety, i.e., LC3-I to LC3-II) that also requires ATG proteins such as ATG4, ATG3, ATG7, and ATG10 (Figure 1; Mizushima, 2020). The local combination of PI3P presence and lipidated LC3-positive subdomains designate the membrane for autophagic activity initiation, heralding phagophore isolation, growth, and closure (Boya et al., 2013).

The membrane remodeling events leading to a dynamic transition from the omegasome to the phagophore are not yet fully deciphered. They probably require the coordination of multiple membrane sources to complete the *de novo* organelle biogenesis. In line with this hypothesis, membrane compartments such as endosomes, Golgi apparatus, ER exit sites, ERGIC vesicles, or plasma membrane have been shown to directly or indirectly participate in autophagosome biogenesis (Molino et al., 2017b). The involvement of ATG9-positive vesicles (which could originate from endosomal and Golgi associated structures) (Hurley and Young, 2017) corroborates the “multimembrane origins” of autophagosomes and suggests that heteromembranous structures are able to interact during phagophore assembly, which has to be tightly regulated in space, a situation that may be favored by the presence of ER membrane subdomains (see section “The ER Membrane and ER Contact Sites in Autophagy Regulation”). Moreover, crucial membrane trafficking regulators such as the recycling endosome-associated small GTPase Rab11 (Puri et al., 2018) are also required at this step, revealing the importance of membrane(s) and proteins classically associated with other trafficking stations in the cell during autophagosome biogenesis and maturation. Finally, the phagophore closes to form the double membrane autophagosome, which will fuse with the lysosome to ensure cargoes degradation and recycling. This fusion step requires regulators associated with the endo-lysosomal pathway, such as SNAREs (including STX proteins) and small Rab GTPases (Molino et al., 2017b).

## The ER and ER Contact Sites in Organelle Biogenesis and Membrane Dynamics

**FIGURE 2:**
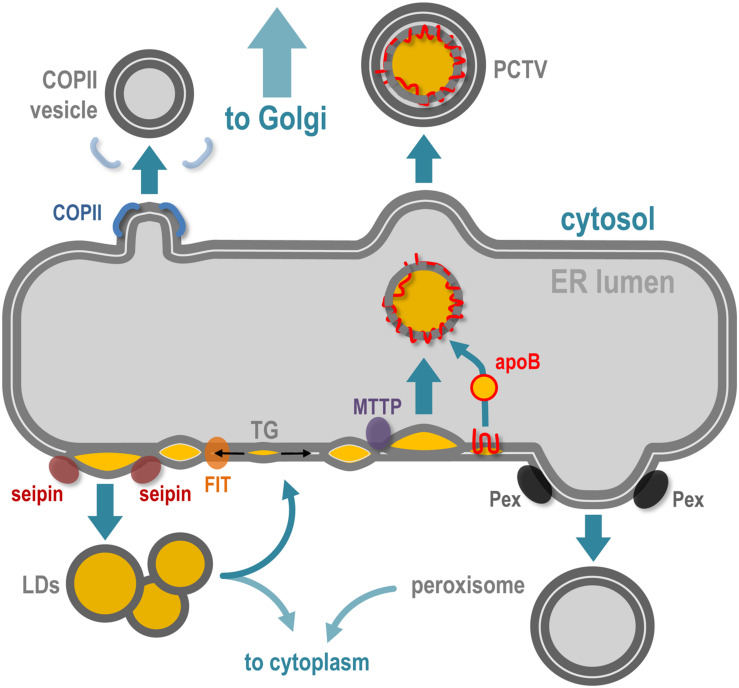
The ER implication in *de novo* membrane-bound structures and organelles. The ER is the specific site of vesicles implied in ER-to-Golgi transport, which notably requires COPII coatomers to deform the ER membrane. Peroxisome biogenesis initiates at the ER membrane via the recruitment of Pex family proteins and the maturation of pre-peroxisome is finalized in the cytoplasm. Accumulation of neutral lipids [triglycerides (TGs)] in the bilayer of the ER leads to ER membrane deformation and release of lipid droplets (LDs) in the cytoplasm, via the coordinated action of FIT and seipin proteins. In specialized cells such as hepatocytes and enterocytes, the ER is also responsible for lipoproteins [chylomicrons in enterocytes and very low density lipoprotein (VLDL) in hepatocytes]. In parallel to LD biogenesis, the TG accumulation in the bilayer leads as well to ER intraluminal budding of neutral lipid structures, via the microsomal triglyceride transfer protein (MTTP), and this structure will be stabilized by fusion with lipidated apoliprotein B (apoB48 in enterocytes and apoB100 in hepatocytes), which leads to pre-lipoprotein synthesis inside the ER lumen. This particle is then transported via the prechylomicron transport vesicle (PCTV) along the secretory pathway via the Golgi apparatus.

The ER is a network of cisternae and tubule-based membrane network, physically connected with the nuclear envelope and the Golgi apparatus, which spreads all over the cytoplasm. One of the major functions of ER is to support membrane protein synthesis and quality control, via ribosomes, regulatory proteins, and proteasomes, as well as posttranslational modifications such as *N*-glycosylation. ER is also a major site of lipid synthesis, notably phospholipids and steroids, and actively participates in Ca^2+^ homeostasis. Besides its key role in protein and lipid synthesis and transport, the ER network is also a platform for *de novo* biogenesis and dynamics of several organelles and membrane structures (Joshi et al., 2017) such as peroxisomes, lipid droplets, and lipoproteins (Figure 2) and membraneless organelles such as P-bodies and stress granules (Lee et al., 2020). In the text that follows I shortly summarize the role of ER in the biogenesis of COPII vesicles, peroxisomes, lipid droplets, and lipoproteins.

## ER and Copii Vesicles

One of the major functions of the ER is to export newly synthesized proteins to the sorting part(s) of the Golgi apparatus to ensure proper vectorized transport of membrane-associated proteins toward the cell plasma membrane and organelles such as the endosomes. ER specialized subdomains, termed ERESs for “ER exit sites,” are involved in the trafficking of protein cargoes en route to the Golgi, a step required for posttranslational modifications as further sorting occurs in Golgi saccules. This ER-to-Golgi vesicular transport is mediated by the small and round COPII vesicles that bud from ERESs in a Sar1 GTPase-mediated mechanism (Peotter et al., 2019). The biogenesis of these COPII vesicles could be regulated by lipids, such as PI4P (phosphatidylinositol-4-phosphate) and dedicated proteins, including the Sec16 oligomers (Joshi et al., 2017).

## ER and Peroxisomes

Peroxisomes are small and transient organelles specialized in metabolism and present in every cell type. One of their key cellular functions is β-oxidation of long-chain fatty acids. The biogenesis and behavior of peroxisomes are directly connected to the cell metabolic state and needs (Smith and Aitchison, 2013). Several recent experimental data strongly suggest that peroxisomes—or pre-peroxysomal structures—originate *de novo* from the ER membrane (and maybe from the mitochondrial membrane as well) and acquire the set of material required for their metabolic functions later, once in the cytoplasm. The budding of pre-peroxysomal vesicles from the ER membrane (Hoepfner et al., 2005) requires several proteins of the Pex family, which might promote the physical formation and detachment of the vesicles, independently of the COP (COPII, COPI) ER and Golgi complexes (Smith and Aitchison, 2013). In a process that requires ubiquitination as well as ATP hydrolysis, Pex proteins cycle between cytosol, ER, and peroxisome membranes to allow their proper targeting and functions at peroxisome surface. Detailed information about peroxisome biogenesis and interplay with ER can be found in the recent review by Mast et al. (2020). Interestingly, peroxisomes are also reported to establish and maintain local tethering with ER membrane (Costello et al., 2017).

## ER and Lipid Droplets

In addition to the transport of cargo proteins and peroxisome biogenesis, ER subdomains contribute to neutral lipids storage and trafficking, mostly via the formation of lipid droplets (LDs), and lipoproteins in specialized cells handling important amounts of lipids (Figure 2). LDs are the main storage organelles for neutral lipids inside the eukaryote cytoplasm (Olzmann and Carvalho, 2019). They are composed of a core of triglycerides (TGs) and esters of cholesterol surrounded by a monolayer of phospholipids, cholesterol and of a variety of proteins, including perilipins. Reflecting their key role in metabolism, LDs are present in every cell type and tissue and are dynamic structures able to interact with many intracellular compartments (Thiele and Spandl, 2008; Gao and Goodman, 2015). LDs can also remain associated with the ER membrane or travel back to it, probably to allow enzyme-mediated local metabolism at the LD–ER interface.

Biogenesis of LD is triggered by neutral lipid synthesis at ER, mostly TG and esters of cholesterol. Local presence of dedicated lipid enzymes, such as diglyceride acyltransferases (DGATs), is thus required for the initiation of LD formation. Neutral lipid hydrophobicity induces dispersion of lipids inside the ER membrane bilayer, and depending on a critical concentration, the neutral lipids will provoke a phase separation that induces the future LD isolation by surrounding phospholipid environment. The accumulation of newly synthesized TG between the two leaflets of the ER phospholipid bilayer is thus considered as the initial trigger (Chapman et al., 2019; Jackson, 2019) that will promote the formation of nascent LDs. The presence of a TG lens inside the ER bilayer promotes the latter deformation toward the cytoplasm side, initiating LD budding at specific sites of the ER membrane, in a surface tension manner (Ben M’barek et al., 2017). While the direct requirement of proteins in the biogenesis of LDs is not completely understood, the physical isolation of LDs from the ER membrane seems to be regulated by ER-associated proteins such as seipin oligomers, LDAF1, perilipins, and FIT proteins, illustrating the very close ER and LD relationship during the biogenesis of the latter (Figure 2). Detailed insights about LD biogenesis can be found in recent reviews (Olzmann and Carvalho, 2019; Renne et al., 2020).

## ER and Lipoproteins Synthesis

In cells managing massive amounts of neutral lipids of alimentary origin, such as enterocytes and hepatocytes, the ER is also a central player in cell protection via the specific biogenesis of lipoproteins, which are mostly composed of apolipoproteins stabilized by neutral lipids, cholesterol, phospholipids, and liposoluble vitamins. The primary lipoproteins synthesized at the ER are the chylomicrons in enterocytes (structurally organized by apob48 in humans) and very large density lipoproteins (VLDL) in hepatocytes (structurally organized by apoB100) (Mansbach and Siddiqi, 2010; Tiwari and Siddiqi, 2012). The apoB protein is synthesized at the ER membrane and, depending on the amount of TG present between the ER membrane leaflets, it will be either stabilized inside the lumen of the ER during its synthesis or retrotranslocated to the ER surface for proteasomal degradation. The process by which the neosynthesized apoB associates with TGs inside the ER lumen depends on the ER-associated proteins PDI and MTTP. The neutral lipids that will engage and stabilize the primordial apoB lipoprotein are of the same origin as LDs, making a strong and dynamic connection between ER membrane, LDs, and lipoproteins (Figure 2; Demignot et al., 2013). The central role of the ER in the management of neutral lipids in enterocytes (Singh et al., 2009; Singh and Cuervo, 2012) is illustrated by a specialized lipophagy (a specialized autophagy aimed toward lipid degradation) that takes place directly at surface of ER membranes in enterocytes facing massive loads of alimentary lipids. In this situation, the biogenesis of autophagosomes occurs concomitantly with the biogenesis of nascent LDs, at the same ER site, to ensure their immediate capture (Khaldoun et al., 2014).

## The ER Membrane and ER Contact Sites in Autophagy Regulation

One of the intriguing features of the organelles formed *de novo* at the ER membrane is that most of them—such as COPII vesicles, LDs, and peroxisomes—are short-lived organelles generated in response to a specific stress or stimulation, which is also true for autophagosome assembly. Thus, the role of the ER in autophagosome biogenesis is particularly interesting because the autophagic program is the major intracellular pathway responding to cellular stress (see section “The Molecular Mechanisms of Autophagy and Autophagosome Biogenesis”) and is thus a key regulator of cellular homeostasis. Indeed, despite the lack of understanding in the detailed steps leading to autophagosome biogenesis, it is now clearly established that ER subdomains are required to initiate the process, in particular omegasome [PI3P enriched ER membrane zone(s)] and ER-mediated contact sites, notably ER–mitochondria and ER–plasma membrane contact sites (MCSs) (Molino et al., 2017a; Prinz et al., 2020).

Membrane contact sites are sites of close apposition between endomembranes. They are considered as molecular hubs for organelle remodeling and membrane dynamics, as well as for metabolite and lipids exchanges and transfer from one compartment to another (Cohen et al., 2018; Scorrano et al., 2019; Prinz et al., 2020). The ER is the central player in MCSs formation and dynamics, as it spreads all through the cytoplasm and would virtually be able to touch all other membrane-bound structures inside the cellular space. ER establishes MCSs with mitochondria, which influences directly the mitochondrial fission/fusion cycles (Friedman et al., 2011), with plasma membrane, peroxisomes, endosomes, lysosomes, LDs, and Golgi (Friedman and Voeltz, 2011; De Matteis and Rega, 2015; Raiborg et al., 2015; Costello et al., 2017; Atakpa et al., 2018).

Interestingly, ER is also engaged in MCSs with several types of endomembranes at the same time, allowing local regulation of membrane-related processes between different organelles, as well as complex signal transduction regulation, particularly at ER–mitochondria MCSs. Complete and updated information about the molecular, physical, and biological features of MCSs are available in several recent detailed reviews (Cohen et al., 2018; Scorrano et al., 2019; Prinz et al., 2020).

**FIGURE 3:**
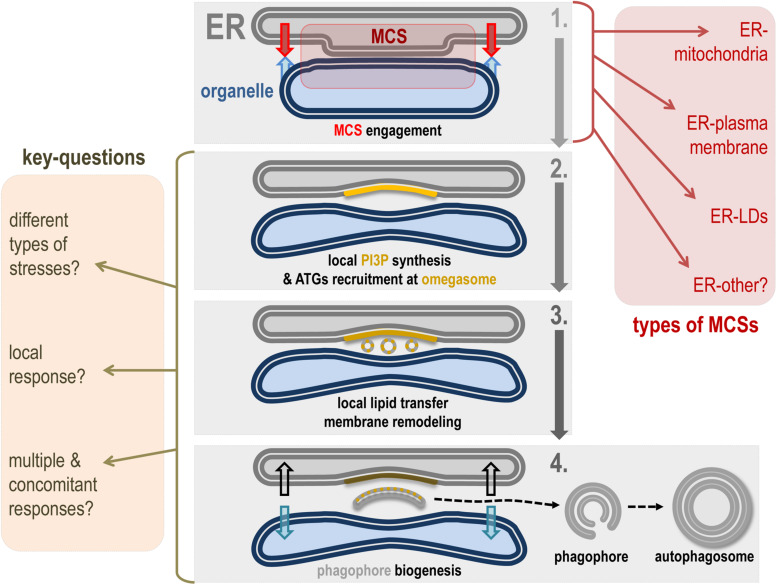
Hypothetical model of ER contact site role in phagophore biogenesis. This scheme represents a hypothetical model of how ER-driven MCSs can be involved in phagophore biogenesis membrane dynamics. Step 1: Under appropriate conditions, the ER membrane mobilizes tethers to ensure the establishment of a MCS with a given membrane. Types of ER-driven MCSs known to participate in autophagosome formation and maturation are listed in the red square (right). Step 2: The specific area created by the ER-engaged MCS allows autophagic machinery mobilization and local PI3P synthesis on the ER subdomain called the omegasome. In turn, the omegasome is considered as a hub for autophagy-associated signaling and membrane dynamics–associated protein interplay. Notably, the PI3P-positive membrane promotes recruitment of PI3P binding proteins directly involved in phagophore assembly. Step 3: The presence of PI3P and associated autophagic membrane modulators as well as the peculiar cytoplasmic properties generated by the MCS space between ER and the engaged organelle allow the *de novo* biogenesis of membrane structures (by direct or indirect lipid transfer and/or capture), which will be necessary for phagophore assembly. Step 4: Newly formed vesicles and/or membrane-bound structures fuse together in the “cradle-like” intermembrane space delineated by the omegasome and this leads to phagophore isolation. At this stage one can imagine that MCS has to be disengaged or turn down to allow the physical disassembly of the phagophore, which will acquire cargoes and specific molecular complexes responsible for its maturation into a closed double membrane autophagosome. The key questions about the role(s) of MCS in autophagosome biogenesis are listed on the yellow square (left).

The phagophore is the first autophagy-related organelle to be formed, prior to autophagosome, in response to a variety of stresses, including nutrient deprivation. The phagophore most probably grows via lipid acquisition and is hallmarked as “future autophagosome” on anchoring lipidated LC3 (see section “The Molecular Mechanisms of Autophagy and Autophagosome Biogenesis”). Despite the identification of most of the regulatory proteins involved in phagophore formation, closure, and maturation, the origin(s) of the membrane(s) that directly participate in its biogenesis is still unclear. In this context, a consensus suggests that the omegasome, an ER membrane transient subdomain, serves as an assembly platform to promote phagophore biogenesis (Ktistakis, 2020). In addition to the omegasome, many endomembranes have been linked directly or indirectly to the phagophore biogenesis: Golgi vesicles, endosomes and endosomal associated vesicles and tubules, mitochondria, lipid droplets, and plasma membrane. Several studies suggest that phagophore/autophagosome biogenesis directly requires ER-driven MCSs, notably ER-mitochondria MCSs, ER–LDs MCSs, and ER–plasma membrane MCSs (Molino et al., 2017a). This variety underlines a multiple and complex lipid sources crosstalk. It is tempting to speculate that the ER/omegasome promotes the necessary condition(s) for *de novo* phagophore assembly and fueling via vesicles (such as ATG9 vesicles) and membrane tubules from diverse origins and spatial localization (Figure 3).

The role of ER–mitochondria MCSs in autophagosome biogenesis in mammalian cells was the first demonstration of the importance of MCs in autophagy (Hamasaki et al., 2013). In a seminal study, the Yoshimori laboratory demonstrated that autophagosomes formed at ER–mitochondria MCSs in response to starvation. The authors showed that the PI3KC3 complex regulatory subunit ATG14L (a key partner of Beclin1 and VPS34 for autophagy-associated PI3P synthesis) relocalized to ER–mitochondria tethering domains together with Stx17, a protein required for membrane remodeling during phagophore and autophagosome biogenesis and maturation (Hamasaki et al., 2013). Other regulators of the PIK3C3 complex such as AMBRA1 are also stabilized at lipid rafts domains localized in the ER–mitochondria MCSs during autophagy (Garofalo et al., 2016). The effect of the ER–mitochondria interface on autophagosome biogenesis was also highlighted by the regulatory role of the VAPB–PTPIP51 tethering complex in autophagy initiation, at least in a non-starvation-induced autophagic program. More precisely, it was shown that the “tethering force” driven by the VAPB–PTPIP51 complex, which regulates membrane-to-membrane contact distance, influences the rate of autophagosome formation at least during non-starvation autophagy induction (Gomez-Suaga et al., 2017).

Recently, ER–plasma membrane tethering factors, and in particular stress responding extended synaptotagmins (E-Syts) (Giordano et al., 2013), were shown to actively participate in phagophore biogenesis in response to a variety of autophagy-associated stresses (Nascimbeni et al., 2017a, b). Membrane tether E-Syt2 interacts transiently with Beclin1 and the autophagy regulatory protein VMP1. This allows recruitment of the PI3KC3 complex at the ER–plasma membrane MCSs, which leads to local PI3P synthesis that initiates the formation of omegasomes harboring key pre-autophagic markers such as the PI3P-binding proteins DCFP1 and WIPI2 (Nascimbeni et al., 2017c).

Intriguingly, the ER seems to initiate (or maintain) a specific membrane tethering situation with the nascent phagophore itself, arguing for a complex membrane interconnection between preexisting ER membrane and newly formed autophagic membrane(s) in time and space. Recent studies show that *de novo* synthesized phospholipids can be directly transferred to the phagophore by fatty acid channeling in a very tight membrane environment (Schütter et al., 2020). ATG2, which might participate in autophagosome biogenesis on the ER–mitochondria interface (Velikkakath et al., 2012; Tang et al., 2019), was recently shown to directly contribute to lipid transfer from the ER membrane to the nascent autophagosome in both yeast and mammalian cells (Kotani et al., 2018; Valverde et al., 2019), in a PI3P-dependent manner (Maeda et al., 2019). ER membrane tethering proteins such as VAP A and VAP B were also suggested to promote phagophore assembly by enhancing and stabilizing local recruitment of the ULK1 signaling complex responsible for PI3KC3 activation, as well as the PI3P-associated WIPI2 targeting to phagophore membrane (Zhao et al., 2018). Whether the ER–phagophore transient tethering is for selective ER membrane degradation by autophagy (a process referred to as ER-phagy) (Khaminets et al., 2015; Dikic, 2018) or for autophagy of ER content such as for pro-collagen degradation (Forrester et al., 2019) is not clear. This underlines the close interplay between membranes that will participate in autophagosome biogenesis and membranes that will be degraded by autophagy.

Interestingly, but making sense with *de novo* phagophore biogenesis from omegasome ER domain, ER and ER–MCS lipid supply associated machineries seem to be central in the initiation of autophagosome biogenesis. While the role of LDs was nicely demonstrated in the early steps of autophagy, notably via delivery of neutral lipids to nascent autophagosomes (Dupont et al., 2014), the ER–LD MCSs can be mobilized to transfer triglycerides and cholesterol esters to the phagophore (Shpilka et al., 2015). A hallmark of the role of MCSs in autophagosome biogenesis is the local PI3P synthesis at these specialized ER domains, both at ER–mitochondria (Hamasaki et al., 2013) and ER–plasma membrane MCSs (Nascimbeni et al., 2017c). Phosphoinositide metabolism is further involved in autophagy through the recruitment of a phosphatidylinositol synthase (Nishimura et al., 2017) on phagophore-forming ER domains, suggesting that local PI synthesis will positively regulate autophagic processes by providing a specific pool of PI ready to be phosphorylated by the PI3KC3 complex. Finally the PI3KC3 partner and autophagic regulator VMP1 (Molejon et al., 2013), which is associated with most of the MCSs in mammalian cells (Tábara and Escalante, 2016), could play a role in the space (i.e., ER-driven MCSs) and time regulation of MCS PI3P-associated synthesis during the autophagic response, as it was recently shown to negatively regulate physical disassociation of phagophores from omegasomes via the Ca^2+^-ATPase SERCA complex (Zhao et al., 2017), demonstrating the importance of Ca^2+^ import from cytosol to ER during autophagy triggering. This highlights the importance of membrane tethering in phagophore assembly, as the absence of MCSs would probably lead to the failure of pre-autophagic machinery recruitment to the ER membrane, while a permanent membrane-to-membrane binding would slow down or abolish the physical separation of the newly formed phagophore from its MCS-associated membrane cradle.

## Concluding Remarks: the ER as a Central Network in the Cellular Stress Response?

ER is not only devoted to protein and lipid biogenesis, but also participates in the regulation of stress(es) sensing signalization hubs (Spang, 2018). This is illustrated by the role of ER–mitochondria MCSs in defense processes such as inflammasome regulation and complex antiviral mechanisms (Namgaladze et al., 2019) that contribute directly to the global cellular stress response and protection. Moreover, ER–mitochondria MCSs have been shown to respond to ER stress, via the unfolded protein response (UPR), PERK signaling, and Ca^2+^/IRE1α signaling (Bravo et al., 2011; van Vliet et al., 2017; Carreras-Sureda et al., 2019) and ER–plasma membrane MCSs are stabilized by several autophagy-inducing stress situations (Nascimbeni et al., 2017c).

The autophagic pathway is a stress response mechanism that undoubtedly mobilizes an important amount of proteins and lipids to maintain cellular homeostasis. *De novo* biogenesis of autophagosomes requires specialized membrane-bound structures assembly and space-and-time coordination for the proper regulation of phagophore formation. ER could be considered as an “organellar and moving scaffold” within the cell, virtually touching every endomembrane in the cytoplasm area, and thus acting as a master regulator of membrane coordination via a wide variety of MCSs. Thus, an important topic of interest for future research on autophagosome biogenesis at ER-driven MCSs will be the analysis of the physical and chemical properties of the cytoplasmic areas of MCSs in comparison with the “classical” cytosol properties, as ATG proteins can organize themselves as liquid-phase condensates to promote pre-autophagosome assembly in yeast (Fujioka et al., 2020). Indeed, MCSs have been associated with non-vesicular lipid transfer (Stefan et al., 2013). This specialized cytosolic microenvironment might promote the conditions required for *de novo* phagophore biogenesis (fusion? exchanges? membrane budding? membrane pinching?). Moreover, the local phosphoinositide metabolism occurring at MCSs (Stefan et al., 2013; Prinz et al., 2020) makes a strong connection with pre-autophagic machinery and membranes mobilization, notably through PI3P and PI4P, two lipids directly associated with autophagic processes and membrane dynamics (Wang et al., 2015; Nascimbeni et al., 2017b, c; Judith et al., 2019).

One of the most intriguing questions on autophagosome biogenesis and ER-driven MCSs concerns the nature of the autophagosomes formed at different MCSs. Are these autophagic structures capturing specific cargoes? Are they responding preferentially to specialized stresses? Are MCSs spatial coordinators (to promote autophagosome biogenesis in a given area of the cell) or “opportunistic” platforms that are randomly mobilized during autophagic processes?

Finally, the identification of other ER MCSs and specific ER MCS-associated tethers (as suggested for VMP1 protein, which plays as well a key function in autophagosome biogenesis regulation) will be one of the challenges in future research on ER membrane mobilization during autophagosome biogenesis.

## Author Contributions

EM wrote the manuscript and prepared the figures.

## Conflict of Interest

The author declares that the research was conducted in the absence of any commercial or financial relationships that could be construed as a potential conflict of interest.
